# Nonlinear Responses and Decoupling Between Soil Organic Carbon Fractions and Extracellular Enzyme Activity Across Salt-Affected Soils of the Qiangtang Plateau

**DOI:** 10.3390/biology15161408

**Published:** 2026-08-17

**Authors:** Chen Chen, Xingyue Li, Shijia Zhou, Hairui Zhao, Mingzhu Cao, Yangong Du, Yarong Chen, Kelong Chen

**Affiliations:** 1 Qinghai Province Key Laboratory of Physical Geography and Environmental Process, College of Geographical Science, Qinghai Normal University, Xining 810008, China; wusaqi321@163.com (C.C.); 18008034655@163.com (X.L.); 18697237052@163.com (H.Z.); cmz6154@163.com (M.C.); cyr0936@163.com (Y.C.); 2 Key Laboratory of Tibetan Plateau Land Surface Processes and Ecological Conservation (Ministry of Education), Qinghai Normal University, Xining 810008, China; 3 National Positioning Observation and Research Station of Qinghai Lake Wetland Ecosystem in Qinghai, National Forestry and Grassland Administration, Haibei 812300, China; 4 Northwest Institute of Plateau Biology, Xining 810008, China; ygdu@nwipb.cas.cn

**Keywords:** alpine salt-affected soils, soil organic carbon fractions, extracellular enzyme activity, XGBoost–SHAP, nonlinear transition points, carbon–enzyme decoupling

## Abstract

Cold alpine salt-affected soils may retain carbon through mineral protection or suppressed microbial decomposition, but these processes remain difficult to distinguish. We measured soil physicochemical properties, four extracellular enzyme activities, soil organic carbon (SOC), and its dissolved (DOC), particulate (POC), and mineral-associated (MAOC) fractions across five soil types on the Qiangtang Plateau. Multivariate analyses and five XGBoost models interpreted using Shapley additive explanations (SHAP) characterized carbon-enzyme associations and identified leading predictors and nonlinear response transition points. Model performance varied among response variables (CV-R^2^ = 0.4094–0.8815). Soda (SD) soil had a distinct carbon-pool composition, and no significant carbon-enzyme correlations remained after Benjamini–Hochberg correction. pH was the leading predictor of SOC and its fractions, whereas total nitrogen and total phosphorus ranked highest for overall enzyme activity. The fitted SHAP contributions changed from negative to positive between pH 8.50 and 9.00 for the carbon variables and at a total nitrogen concentration of 0.71 g·kg^−1^ for enzyme activity. These model-dependent transition points indicate that carbon-pool restructuring and nutrient-related changes in enzyme activity occur over different environmental ranges. SD soil contained the highest carbon concentrations but the smallest MAOC proportion and the largest POC and DOC proportions, indicating relative enrichment of labile carbon fractions. By jointly analyzing carbon fractions and enzyme activity within an interpretable XGBoost-SHAP framework, this study helps distinguish high carbon stocks from stable sequestration and shows why total SOC alone may overestimate long-term carbon stability in alpine salt-affected soils.

## Simple Summary

Alpine soils store large amounts of carbon, but strong evaporation can concentrate salts and increase soil alkalinity. We examined five soil types affected by salt and alkalinity on the Qiangtang Plateau to determine how soil conditions and microbial enzymes relate to different forms of stored carbon. These enzymes help break down organic matter. Carbonate-rich soda soil contained the most carbon, but much of it was dissolved in soil water or held in loose particles rather than bound to minerals. Mineral-bound carbon is generally more stable. After accounting for multiple comparisons, no reliable relationship between carbon forms and enzyme activity remained in soda soil. Across the five soil types, carbon forms were related mainly to soil alkalinity, whereas enzyme activity was more closely related to nitrogen and phosphorus availability. Carbon storage and microbial breakdown therefore did not change together. These findings show that high carbon content does not necessarily indicate stable, long-term storage. Assessments based only on total soil carbon may therefore overestimate carbon stability. Considering carbon form, nutrient availability, and microbial activity can improve estimates of carbon retention and loss risk in fragile plateau ecosystems under climate change.

## 1. Introduction

Soils are a major carbon reservoir in terrestrial ecosystems, storing approximately twice as much carbon as the atmosphere and three times as much as vegetation [1]. Soil organic carbon (SOC) is an important component of this reservoir, and even small changes can substantially affect the global carbon cycle and atmospheric greenhouse gas concentrations [2]. However, SOC is not homogeneous because its fractions differ markedly in origin, bioavailability, turnover rate, and stabilization mechanism [3,4,5]. Total SOC alone therefore cannot reliably indicate carbon-pool stability or distinguish the short-term accumulation of labile carbon from the long-term sequestration of stable carbon.

Continued warming and increasing evaporation are altering water and salt balances in alpine wetlands, potentially increasing the risk of soil salinization and alkalization [6]. Salinization and alkalization can reduce plant-derived carbon inputs while also constraining microbial decomposition. Lower plant productivity limits carbon inputs, whereas high salinity, ion toxicity, and elevated pH can suppress microbial metabolism and extracellular enzyme activity, thereby slowing organic carbon decomposition [7,8]. A global model estimated an average organic carbon loss of approximately 3.47 t·ha^−1^ following salinization [8]. However, when reduced plant inputs were excluded and only decomposition inhibition was considered, the model predicted an increase of approximately 0.48 t·ha^−1^ [8]. Incubation and regional studies likewise show that increasing salinity generally suppresses carbon mineralization. The magnitude of this effect depends on salinity, soil texture, and microbial community composition and may vary nonlinearly [9,10,11]. Severe salinity and alkalinity also restrict microbial carbon use and reduce several soil enzyme activities [11,12,13]. The net carbon response therefore depends on the balance between reduced plant inputs and inhibited decomposition. Current evidence does not establish whether salinization ultimately causes carbon loss or retention [7,8,12].

Most studies assess these responses using total SOC, CO_2_ fluxes, or individual enzyme activities [3,4,7]. These indicators cannot distinguish stable carbon sequestration from the retention of labile carbon when decomposition is constrained. Single-factor correlations and linear models also have limited capacity to resolve the relative contributions, interactions, and nonlinear responses of correlated environmental variables [14]. We therefore combine XGBoost, which captures nonlinear effects and predictor interactions without prespecifying their functional forms, with SHAP, which quantifies the magnitude and direction of each predictor’s contribution. Recent applications of Shapley values to SOC prediction and machine-learning analyses of nonlinear microbial responses support this approach [15,16].

The Qiangtang Plateau in Tibet, China, is one of the world’s highest inland alpine arid regions. Its harsh climate and heterogeneous salt composition have produced alpine ecosystems containing several types of salt-affected soil. Compared with lowland and coastal salt-affected soils, SOC turnover in this region is persistently constrained by low temperature, aridity, and nutrient limitation. Spatial variation in salt ion composition and salt-affected soil type further complicates carbon-pool formation and stabilization [17,18,19]. Studies of salt-affected croplands, inland plains, and coastal tidal wetlands show that salinity affects microbial decomposition, extracellular enzyme activity, and SOC storage [7,9,11,13]. However, the temperature, moisture, and soil salt conditions of these regions differ markedly from those of the Qiangtang Plateau. Whether their findings apply to alpine habitats where cold, aridity, salinity, and alkalinity co-occur remains unclear. Research on the Tibetan Plateau has separately examined grassland degradation and restoration [20,21], soil respiration [22], and the effects of climate and weathering on organic carbon fractions [23,24]. Regional evidence remains insufficient to determine whether salt-affected soil types alter carbon-pool composition and carbon-enzyme associations. It is also unclear whether SOC, its fractions, and extracellular enzyme activities respond similarly to environmental conditions.

Against this background, we examine five representative salt-affected soil types on the Qiangtang Plateau. In this study, carbon-enzyme decoupling refers to reduced coordination between organic carbon fractions and extracellular enzyme activities. Operational evidence for decoupling includes divergence in correlation direction, fewer statistically supported carbon-enzyme associations, or different leading environmental predictors for carbon fractions and geometric mean enzyme activity (GMEA). We hypothesize that carbon-pool composition and carbon-enzyme association patterns differ among salt-affected soil types. We further expect carbon fractions to respond mainly to alkalinity, whereas GMEA responds more strongly to nutrient availability, with both showing nonlinear response transition points. To test these hypotheses, we measure soil physicochemical properties, four extracellular enzyme activities, total SOC, dissolved organic carbon (DOC), particulate organic carbon (POC), and mineral-associated organic carbon (MAOC). We combine PCoA, within-soil-type Pearson correlation analysis with Benjamini–Hochberg correction, and XGBoost–SHAP modelling to compare carbon-pool composition, evaluate carbon–enzyme associations, and identify major predictors and response transition points. The methodological contribution of this study is the joint analysis of operationally defined carbon fractions and multiple extracellular enzyme activities across chemically distinct alpine salt-affected soils. Interpretable XGBoost-SHAP models link these measurements to predictor importance, response direction, and nonlinear transition points within a single analytical framework.

## 2. Materials and Methods

### 2.1. Study Area

**Figure 1:**
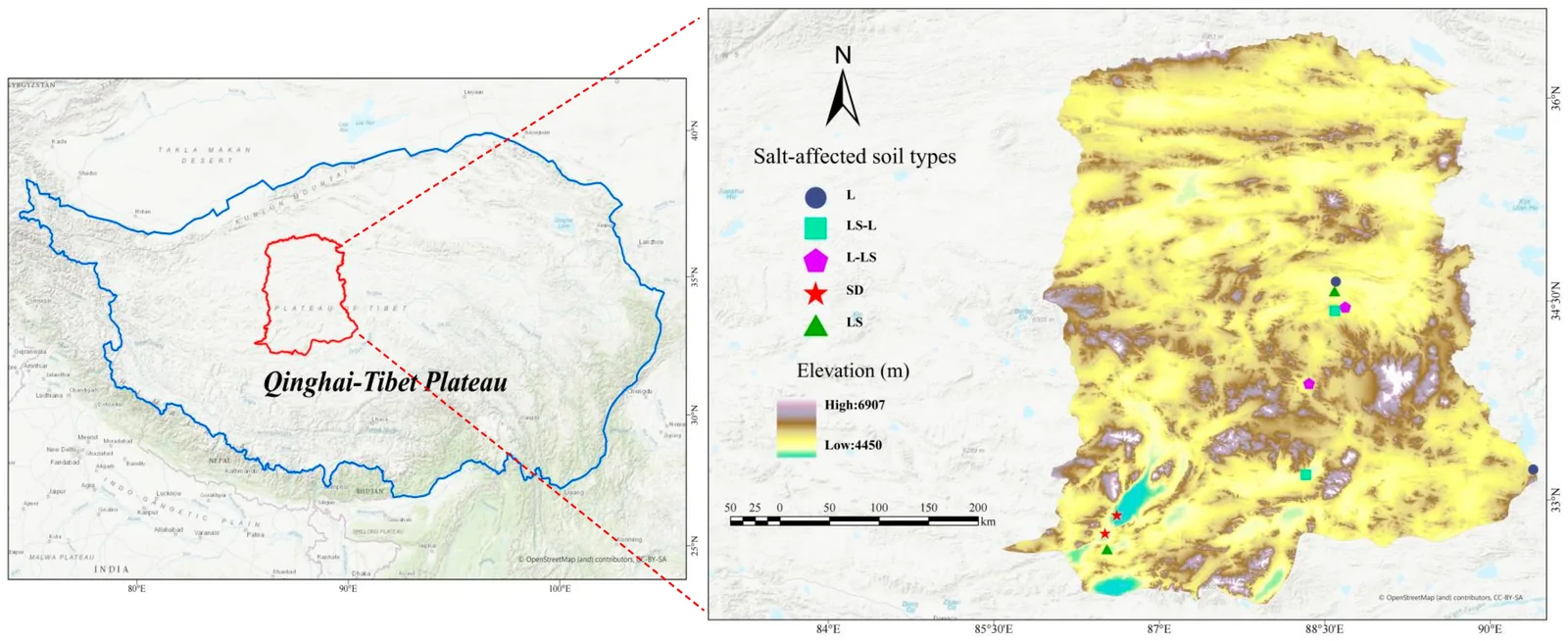
Location of the study area and distribution of sampling sites for the five salt-affected soil types on the Qiangtang Plateau.

The study area is located in the southern Qiangtang Plateau in the central Tibetan Plateau, between 32°32′08″ and 34°38′35″ N and between 86°15′21″ and 90°23′26″ E. The sampling sites are mainly distributed across western Nagqu City and the southern margin of Nima County (Figure 1). Western Nagqu City has a plateau subfrigid semi-arid climate. The mean annual temperature ranges from −2.0 to −0.5 °C, and annual precipitation ranges from 200 to 300 mm. Annual evaporation ranges from 1500 to 2000 mm, and annual sunshine duration ranges from 2800 to 3000 h. Frost may occur throughout the year, and the area has no distinct frost-free period. The southern margin of Nima County lies along the southern boundary of the uninhabited Qiangtang region and has a plateau frigid arid climate. This area is characterized by low precipitation, high evaporation, and frequent strong winds. The mean annual temperature ranges from −4.0 to −2.0 °C. The extreme maximum temperature reaches 18 °C, whereas the extreme minimum temperature falls below −45 °C. Annual precipitation ranges from 150 to 200 mm, and annual evaporation ranges from 2000 to 2500 mm. Annual sunshine duration exceeds 3000 h, strong winds occur on more than 100 d each year, and the area has no distinct frost-free period.

### 2.2. Classification Criteria for Salt-Affected Soils

The classification of salt-affected soils in China is based mainly on the ratios of the milliequivalent concentrations of water-soluble anions in soil extracts. Following Wang et al. [25], soils are classified according to the equivalent ratio of Cl^−^ to SO_4_^2−^. A Cl^−^/SO_4_^2−^ ratio > 2 indicates the chloride type (L), while a ratio from 1 to 2 indicates the sulfate-chloride type (LS-L). A ratio from 0.5 to 1 indicates the chloride-sulfate type (L-LS), while a ratio <0.5 indicates the sulfate type (LS). Soils in which CO_3_^2−^ is the dominant measured water-soluble anion and the pH is high are classified as the soda type (SD). The equivalent ratios are calculated as follows:

$$\mathrm{Ratio}=\frac{C_{{\mathrm{Cl}}^{-}}/35.5}{C_{{\mathrm{SO}}_{4}^{2-}}/48}$$

where $C_{{\mathrm{Cl}}^{-}}$ and $C_{{\mathrm{SO}}_{4}^{2-}}$ represent the measured contents of Cl^−^ and SO_4_^2−^, respectively (mg·g^−1^ on a dry-soil basis); 35.5 and 48 are their respective equivalent masses; and Ratio is the dimensionless equivalent ratio of Cl^−^ to SO_4_^2−^.The vegetation characteristics and major water-soluble anion contents of the sampling sites are summarized in Table 1.

### 2.3. Soil Sampling

Sampling was conducted from mid- to late August 2025. Candidate locations were randomly selected within accessible parts of the study area without prior assignment to a salt-affected soil type. After sampling, sites were classified according to their water-soluble anion composition using the criteria described in Section 2.2. Because poor road accessibility limited the number of feasible locations, two geographically distinct sites were retained for each soil type to maintain balanced sampling effort. The resulting 10 sites represented five soil types: chloride (L), chloride-sulfate (L-LS), sulfate-chloride (LS-L), sulfate (LS), and soda (SD). The two sites per soil type represented the available site-level spatial replication. Within each site, three 1 m × 1 m quadrats were established along a diagonal as nested subsamples to characterize within-site heterogeneity. After removing surface litter, soil was collected separately from the 0–10 and 10–20 cm layers of each quadrat using a soil auger. This design yielded 60 soil-layer observations nested within 30 quadrats and 10 sites. Undisturbed soil cores were collected adjacent to each quadrat at the corresponding depths using 100 cm^3^ core rings for bulk density measurement.

Soil samples were transported promptly to the laboratory. Visible plant residues, roots, and gravel were removed before subsampling. Samples used for extracellular enzyme assays were passed through a 2 mm sieve, stored at 4 °C, and analyzed within 7 d of collection. Samples used to determine pH, electrical conductivity, available phosphorus, and dissolved organic carbon were air-dried at room temperature and passed through a 2 mm sieve. Samples used for particulate organic carbon and mineral-associated organic carbon fractionation were air-dried and passed through a 2 mm sieve without further grinding to preserve aggregate structure. Air-dried samples used to determine soil organic carbon, total nitrogen, total phosphorus, and total potassium were further ground and passed through a 0.25 mm sieve.

### 2.4. Measurement of Soil Physicochemical Properties, Organic Carbon Fractions, and Enzyme Activities

Soil bulk density (BD) was measured using the soil core method [26]. Soil pH was measured in a 1:2.5 soil-to-water suspension using a PHS-3C pH meter(Shanghai INESA Scientific Instrument Co., Ltd., Shanghai, China). Electrical conductivity was measured in a 1:5 soil-to-water extract according to HJ 802-2016 and is reported as EC_1_:_5_ (hereafter EC). Because international salinity classes are generally based on the electrical conductivity of saturated-paste extracts (ECe), EC_1_:_5_ was used only for relative comparisons among soil types and was not converted to ECe or used to assign salinity severity classes. Available phosphorus (AP) was extracted with 0.5 mol·L^−1^ NaHCO_3_ and measured by molybdenum-antimony spectrophotometry. Total nitrogen (TN) was measured using the Kjeldahl method according to HJ 717-2014 [27]. Total phosphorus (TP) was determined by alkali fusion followed by spectrophotometry according to HJ 632-2011, and total potassium (TK) was measured by flame photometry after HNO_3_-HClO_4_-HF digestion. Soil organic carbon (SOC) was measured by potassium dichromate oxidation with external heating. Dissolved organic carbon (DOC) was extracted with cold water at a soil-to-water ratio of 1:5, filtered through a 0.45 μm membrane and analyzed using a TOC-L total organic carbon analyzer(Shimadzu Corporation, Kyoto, Japan). Particulate organic carbon (POC) and mineral-associated organic carbon (MAOC) were separated by dispersing soil samples in 5 g·L^−1^ sodium hexametaphosphate for 18 h and passing the suspension through a 53 μm sieve. The retained and passing fractions were operationally defined as POC and MAOC, respectively, and their carbon contents were measured by potassium dichromate oxidation followed by titration. Urease (URE), sucrase (SUC), β-glucosidase (BG), and catalase (CAT) activities were measured using sodium phenolate-sodium hypochlorite colorimetry, 3,5-dinitrosalicylic acid colorimetry, a fluorometric microplate assay, and a microassay kit, respectively. BG and SUC were selected to represent carbohydrate hydrolysis, URE to represent nitrogen transformation through urea hydrolysis, and CAT to represent the oxidative stress response. Because no phosphorus-acquiring enzyme was measured, this enzyme panel was intended to characterize complementary hydrolytic and oxidative functions rather than complete carbon, nitrogen, and phosphorus acquisition stoichiometry. Water-soluble CO_3_^2−^, Cl^−^, and SO_4_^2−^ were extracted at a soil-to-water ratio of 1:5 and determined by ion chromatography.

### 2.5. Data Analysis

#### 2.5.1. Statistical and Multivariate Analyses

All data processing, statistical analyses, and visualization were conducted in R version 4.5.2. Results were presented as the mean ± standard error. Univariate analyses used quadrat-level measurements, with soil depth treated as a paired factor within each quadrat. The quadrats were nested within sites, but site-level clustering was not modeled separately. The resulting statistical comparisons therefore described patterns among the sampled sites and were not intended to support unrestricted regional extrapolation. Normality and homogeneity of variance were assessed using the Shapiro–Wilk and Levene’s tests, respectively. Variables meeting both assumptions were analyzed using paired-samples t-tests to compare the 0–10 and 10–20 cm layers within the same quadrat. One-way ANOVA followed by Tukey’s honestly significant difference test was used to compare soil physicochemical properties, organic carbon fractions, and extracellular enzyme activities among soil types within each soil layer. Two-way ANOVA was used to evaluate the effects of soil type, soil depth, and their interaction on each variable. Statistical significance was set at *p* < 0.05. These analyses were performed using the stats, car, and agricolae packages.

Before calculating the relative distribution of the measured carbon fractions, DOC concentrations were converted from mg·kg^−1^ to g·kg^−1^. The relative proportion of each fraction was calculated by dividing its concentration by the summed concentrations of DOC, POC, and MAOC and multiplying by 100. Principal coordinates analysis (PCoA) based on Bray–Curtis dissimilarity was used to characterize variation in SOC and its measured fractions among soil types. Differences in carbon-pool composition were tested using permutational multivariate analysis of variance (PERMANOVA) with the adonis2 function in the vegan package.

Within each soil type, two-sided Pearson correlations were calculated between seven physicochemical properties (BD, EC, AP, TP, TK, TN, and pH) and eight carbon or enzyme variables (SOC, DOC, POC, MAOC, CAT, BG, SUC, and URE), as well as among the eight carbon or enzyme variables. Each soil type included 12 soil-layer observations nested within six quadrats and two sites. The Benjamini–Hochberg procedure was applied separately within each soil type to the 56 correlations between physicochemical properties and carbon or enzyme variables and the 28 pairwise correlations among carbon fractions and enzyme activities. Associations with a BH-adjusted *p* < 0.05 were considered significant. Correlation networks and other figures were produced using ggplot2 and patchwork.

#### 2.5.2. XGBoost-SHAP Analysis

To integrate the four extracellular enzyme activities measured on different scales, the activity of each enzyme was first normalized by its mean across all samples:

$$\left(E_{rel,i}=E_{i}/\bar{E}\right)$$

where $E_{i}$ is the measured activity of enzyme *E* in sample *i*, and $\bar{E}$ is the mean activity of that enzyme across all samples. The geometric mean enzyme activity (GMEA) was then calculated as:

$$\left(GMEA_{i}=(BG_{rel,i}\times URE_{rel,i}\times SUC_{rel,i}\times CAT_{rel,i}{)}^{1/4}\right)$$

where $BG_{rel,i}$, $URE_{rel,i}$, $SUC_{rel,i}$, and $CAT_{rel,i}$ represent the mean-normalized activities of β-glucosidase, urease, sucrase, and catalase in sample *i*, respectively. Mean normalization produced positive, dimensionless values and placed the four enzyme activities on comparable scales. GMEA was treated as a composite index of the four measured enzyme activities rather than a comprehensive measure of microbial carbon, nitrogen, and phosphorus acquisition.

Five gradient-boosted tree regression models were fitted using XGBoost. BD, EC, pH, AP, TP, TK, and TN were used to predict GMEA. The same variables, together with GMEA, were then used in four separate models for SOC, DOC, POC, and MAOC. Pairwise Spearman correlations were examined among predictors within each model. Because all absolute correlation coefficients were below 0.70, no predictors were excluded because of pairwise collinearity.

Given the sample size, we used a fixed, low-complexity parameter set instead of an extensive hyperparameter search. All models used a squared-error regression objective, a learning rate (eta) of 0.10, a maximum tree depth of 4, subsample and column-subsample ratios of 0.80, and 100 boosting rounds. These settings were selected to limit model complexity and reduce overfitting. The random seed was set to 42 to ensure reproducibility. Predictive performance was evaluated by fivefold cross-validation using CV-R^2^ and CV-RMSE (Table A1). The models were used to characterize predictive relationships within the observed environmental range, without extrapolation beyond the study dataset. All analyses were conducted in R version 4.5.2 using the xgboost and SHAPforxgboost packages.

Shapley additive explanations (SHAP) were used to quantify the contribution of each predictor to the model output. Positive SHAP values indicated an increase in the predicted value relative to the model baseline, whereas negative values indicated a decrease. Global predictive importance was calculated as the mean absolute SHAP value of each predictor across all samples. Relative predictive importance was obtained by dividing each mean absolute SHAP value by the sum across all predictors and multiplying by 100.

LOESS was fitted to the SHAP dependence data using a span of 0.75. Approximate 95% confidence bands were displayed to represent uncertainty in the fitted curves. A response transition point was defined as the unique crossing at which the fitted SHAP contribution changed from negative to positive. The 25th and 75th percentiles indicated the central range of the observed predictor values, while interpretation was limited to the full observed range. To assess sensitivity to the smoothing parameter, the LOESS curves were refitted using spans of 0.60, 0.75, and 0.90. The XGBoost models and sample-level SHAP values were held constant so that this analysis isolated the effect of LOESS smoothing. Transition points were treated as descriptive, model-dependent features of the dataset rather than universal ecological thresholds. The confidence bands quantify uncertainty in the fitted curves and do not represent confidence intervals for the transition points.

## 3. Results

### 3.1. Soil Organic Carbon Pools and Fractions Across Salt-Affected Soil Types

**Figure 2:**
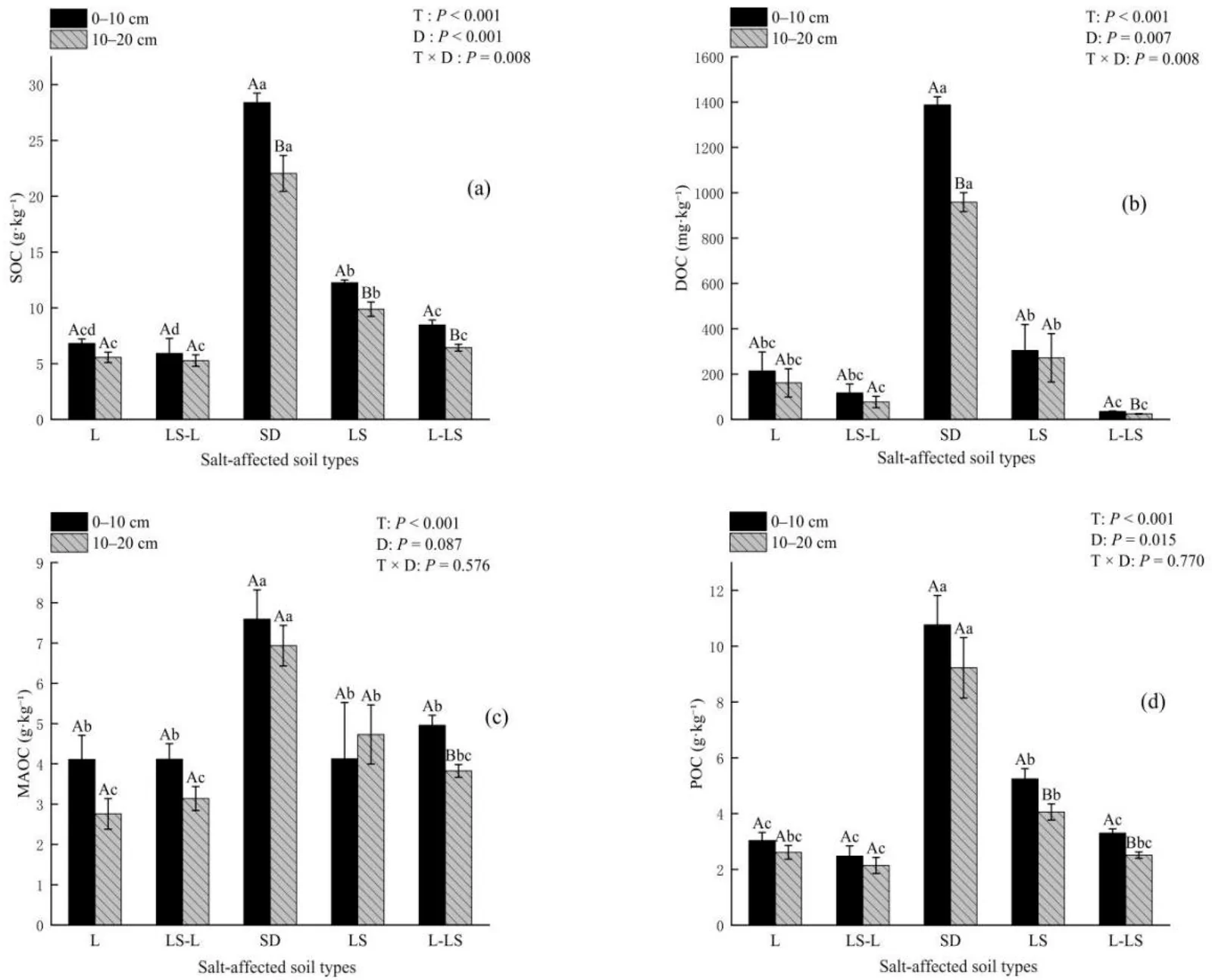
Soil organic carbon and its fractions across salt-affected soil types. Panels (**a**–**d**) show SOC, DOC, MAOC, and POC, respectively. Values are means ± SE. Different lowercase letters indicate differences among soil types within the same depth, whereas different uppercase letters indicate differences between depths within the same soil type (*p* < 0.05). T, D, and T × D denote soil type, soil depth, and their interaction, respectively.

The physicochemical characteristics of the five salt-affected soil types were summarized in Table A2. SD soil had the highest pH and relatively low TN and TP contents. Soil type significantly affected SOC, DOC, POC, and MAOC concentrations (*p* < 0.001). Soil depth significantly affected SOC, DOC, and POC concentrations (*p* < 0.05) but had no significant overall effect on MAOC (*p* = 0.087). The interaction between soil type and soil depth was significant only for SOC and DOC (*p* < 0.01; Figure 2a,b).

SD soil had significantly higher SOC and organic carbon fraction concentrations than the other soil types, with the highest values observed in the surface layer. Surface-layer SOC, DOC, MAOC, and POC concentrations reached 28.39 g·kg^−1^, 1387.64 mg·kg^−1^, 7.59 g·kg^−1^, and 10.77 g·kg^−1^, respectively (Figure 2a–d). The depth-related differences in SOC and DOC were most pronounced in SD soil, where their concentrations decreased by 22.3% and 30.9%, respectively, from the surface to the subsurface layer (*p* < 0.01; Figure 2a,b). Changes between soil layers were smaller in the other soil types. Across all soil types, POC concentration was higher in the surface layer than in the subsurface layer (*p* = 0.015; Figure 2d). The lowest POC concentration occurred in LS-L soil, at 2.30 g·kg^−1^. MAOC differed significantly between soil layers only in L-LS soil, with no significant depth-related differences detected in the other soil types (Figure 2c).

**Figure 3:**
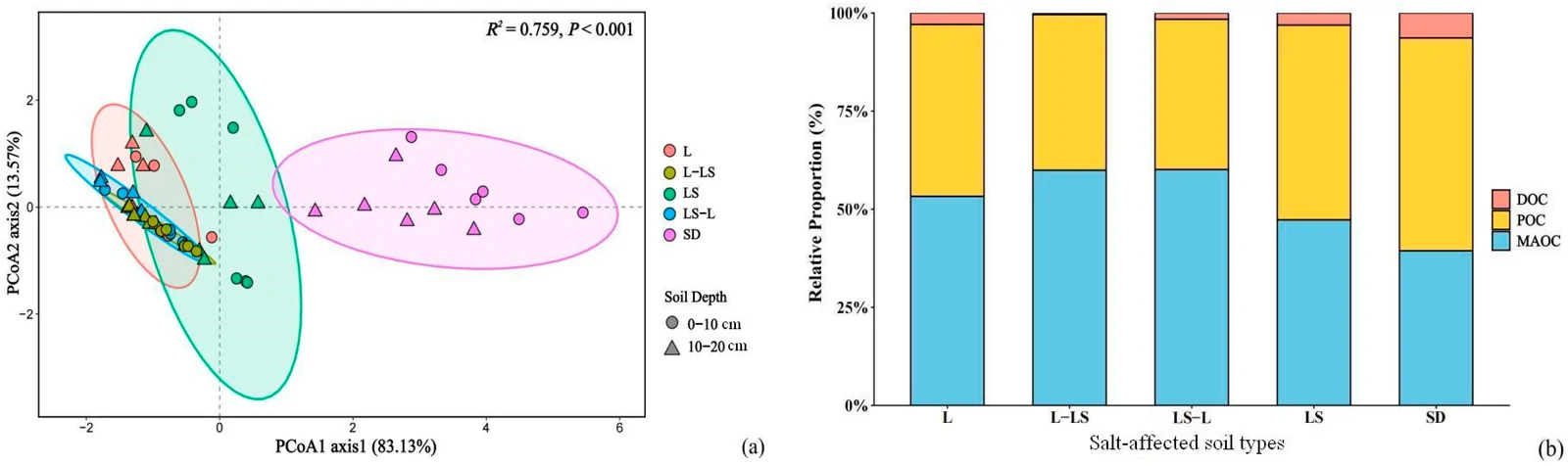
Composition and relative distribution of soil organic carbon fractions across salt-affected soil types. (**a**) Principal coordinates analysis (PCoA) of soil organic carbon and its fractions based on Bray-Curtis dissimilarity. Colors and symbols represent soil types and soil depths, respectively, and ellipses indicate the 95% confidence intervals for each soil type. The R^2^ and *p* values are obtained from permutational multivariate analysis of variance (PERMANOVA). (**b**) Relative proportions of DOC, POC, and MAOC in the sum of the three measured organic carbon fractions (%). SOC, soil organic carbon; DOC, dissolved organic carbon; POC, particulate organic carbon; MAOC, mineral-associated organic carbon.

The composition of soil organic carbon fractions differed significantly among salt-affected soil types (PERMANOVA, R^2^ = 0.759, *p* < 0.001; Figure 3a). The first two principal coordinate axes together explained 96.7% of the total variation. SD samples formed a distinct cluster and were clearly separated from the L, L-LS, LS-L, and LS samples in the ordination space. Within the measured carbon-fraction pool, MAOC accounted for 53.28%, 59.93%, and 60.12% in L, L-LS, and LS-L soils, respectively. Its relative contribution decreased to 47.27% in LS soil and reached its lowest value of 39.41% in SD soil (Figure 3b). The relative contributions of DOC and POC were higher in LS and SD soils than in the other soil types and reached their highest values in SD soil, at 6.36% and 54.23%, respectively.

### 3.2. Relationships Among Soil Organic Carbon Fractions, Soil Properties, and Enzyme Activities Across Salt-Affected Soil Types

**Figure 4:**
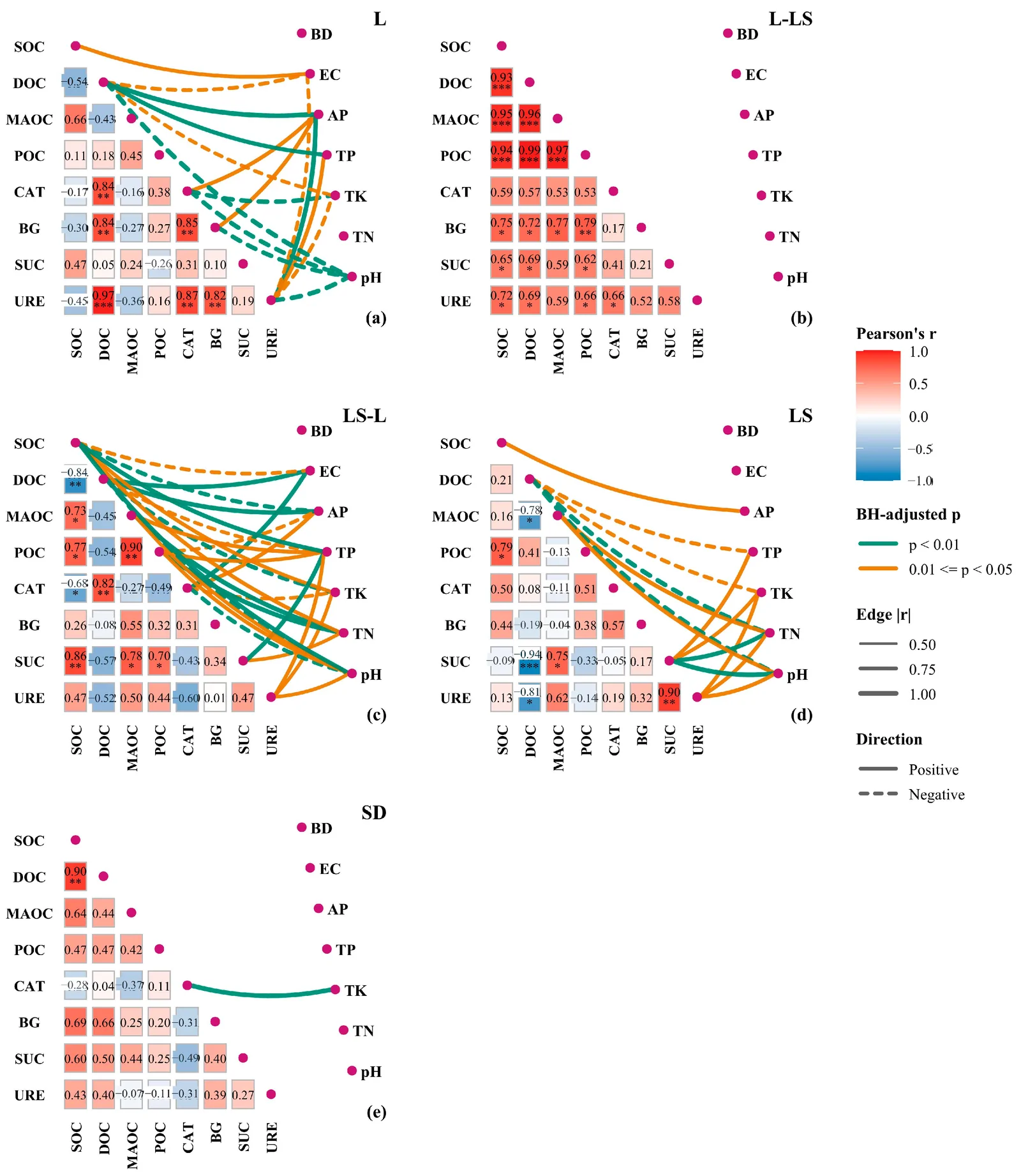
Pearson correlations among soil properties, organic carbon fractions, and extracellular enzyme activities in (**a**) chloride (L), (**b**) chloride-sulfate (L-LS), (**c**) sulfate-chloride (LS-L), (**d**) sulfate (LS), and (**e**) soda (SD) soils. Matrices show Pearson’s r among carbon fractions and enzyme activities. Edges show correlations of soil properties with carbon fractions or enzyme activities; line type, width, and color represent direction, |r|, and BH-adjusted significance, respectively. *, **, and *** indicate BH-adjusted *p* < 0.05, *p* < 0.01, and *p* < 0.001. Green and orange edges indicate *p* < 0.01 and 0.01 ≤ *p* < 0.05, respectively. BD, bulk density; EC, electrical conductivity; AP, available phosphorus; TP, total phosphorus; TK, total potassium; TN, total nitrogen; SOC, soil organic carbon; DOC, dissolved organic carbon; POC, particulate organic carbon; MAOC, mineral-associated organic carbon; CAT, catalase; BG, β-glucosidase; SUC, sucrase; URE, urease.

After BH correction, LS-L soil retained the largest number of significant correlations between physicochemical properties and carbon or enzyme variables (27), followed by L (16) and LS soils (14; Figure 4). No significant correlations within this set remained in L-LS soil, whereas SD soil retained only a positive correlation between TK and CAT. In L soil, AP and TP were positively correlated with DOC and selected enzyme activities, whereas pH and TK were negatively correlated with DOC and several enzymes. In LS soil, pH and TN were negatively correlated with DOC but positively correlated with MAOC, SUC, and URE. In LS-L soil, the correlations of pH, TN, TP, TK, AP, and EC with carbon fractions and enzyme activities varied in direction.

Correlations among carbon fractions and enzyme activities also differed among soil types. In L soil, DOC was positively correlated with CAT, BG, and URE (r = 0.84–0.97, BH-adjusted *p* < 0.01). L-LS soil retained 17 significant correlations, including positive correlations among all carbon fractions (r = 0.93–0.99, BH-adjusted *p* < 0.001) and several positive carbon–enzyme correlations. In LS soil, DOC was negatively correlated with SUC and URE, whereas MAOC was positively correlated with SUC. LS-L soil contained both positive and negative carbon–enzyme correlations. In SD soil, SOC and DOC were positively correlated, but no significant correlation between any carbon fraction and enzyme activity remained after BH correction.

### 3.3. Key Predictors and Nonlinear Response Transition Points Identified by XGBoost–SHAP

**Figure 5:**
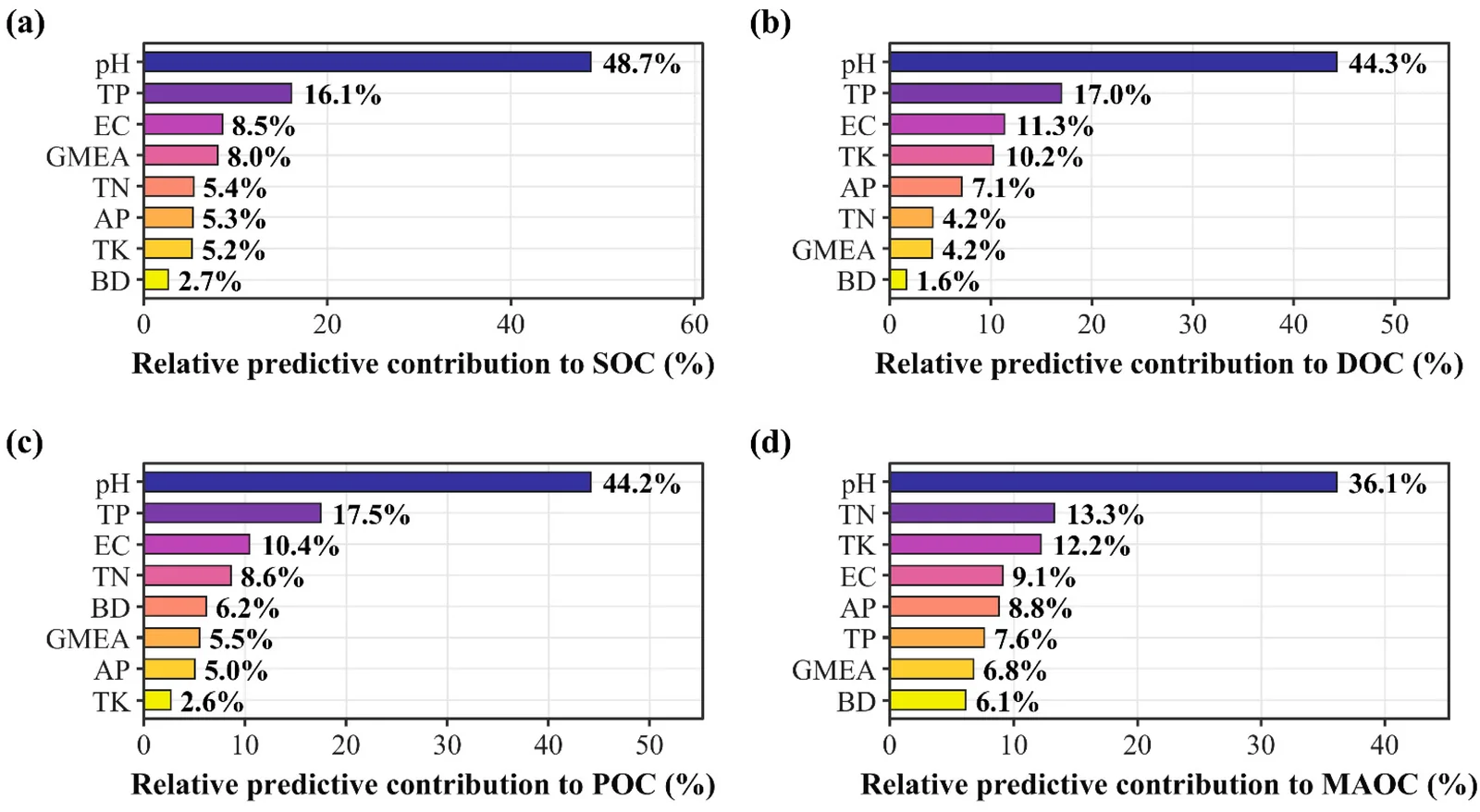
Relative predictive importance of variables in the XGBoost-SHAP models for (**a**) SOC, (**b**) DOC, (**c**) POC, and (**d**) MAOC. Percentages are normalized mean absolute SHAP values, with larger values indicating greater contributions to the corresponding model prediction. GMEA, geometric mean enzyme activity.

**Figure 6:**
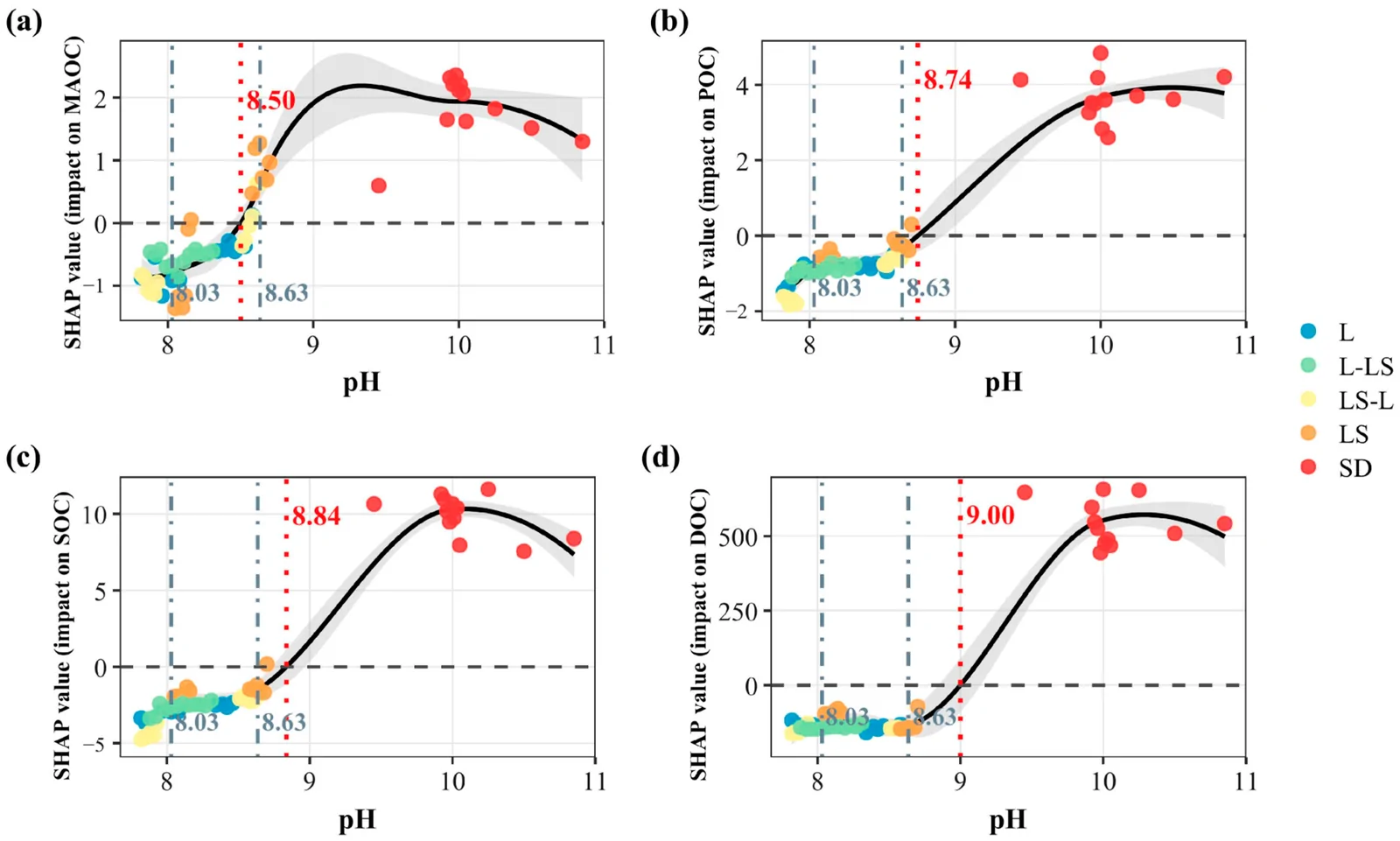
SHAP dependence of pH in the models for (**a**) MAOC, (**b**) POC, (**c**) SOC, and (**d**) DOC. Black curves and gray bands show LOESS fits and 95% confidence intervals, respectively. SHAP values indicate positive or negative contributions to model predictions relative to the baseline. Red lines mark zero-crossing transition points, and blue-gray lines indicate the 25th and 75th percentiles of pH.

Across the five XGBoost models, CV-R^2^ values ranged from 0.4094 to 0.8815, and the corresponding CV-RMSE values were reported in Table A1. pH had the greatest relative predictive importance for SOC, DOC, POC, and MAOC, with contributions of 48.7%, 44.3%, 44.2%, and 36.1%, respectively (Figure 5). TP ranked second for SOC, DOC, and POC, accounting for 16.1–17.5% of relative predictive importance. For MAOC, TN and TK ranked second and third, with contributions of 13.3% and 12.2%, respectively. The SHAP dependence curves showed that the fitted pH contributions to MAOC, POC, SOC, and DOC crossed zero at approximately 8.50, 8.74, 8.84, and 9.00, respectively (Figure 6). The fitted contribution of pH was predominantly negative below each transition point and positive above it.

**Figure 7:**
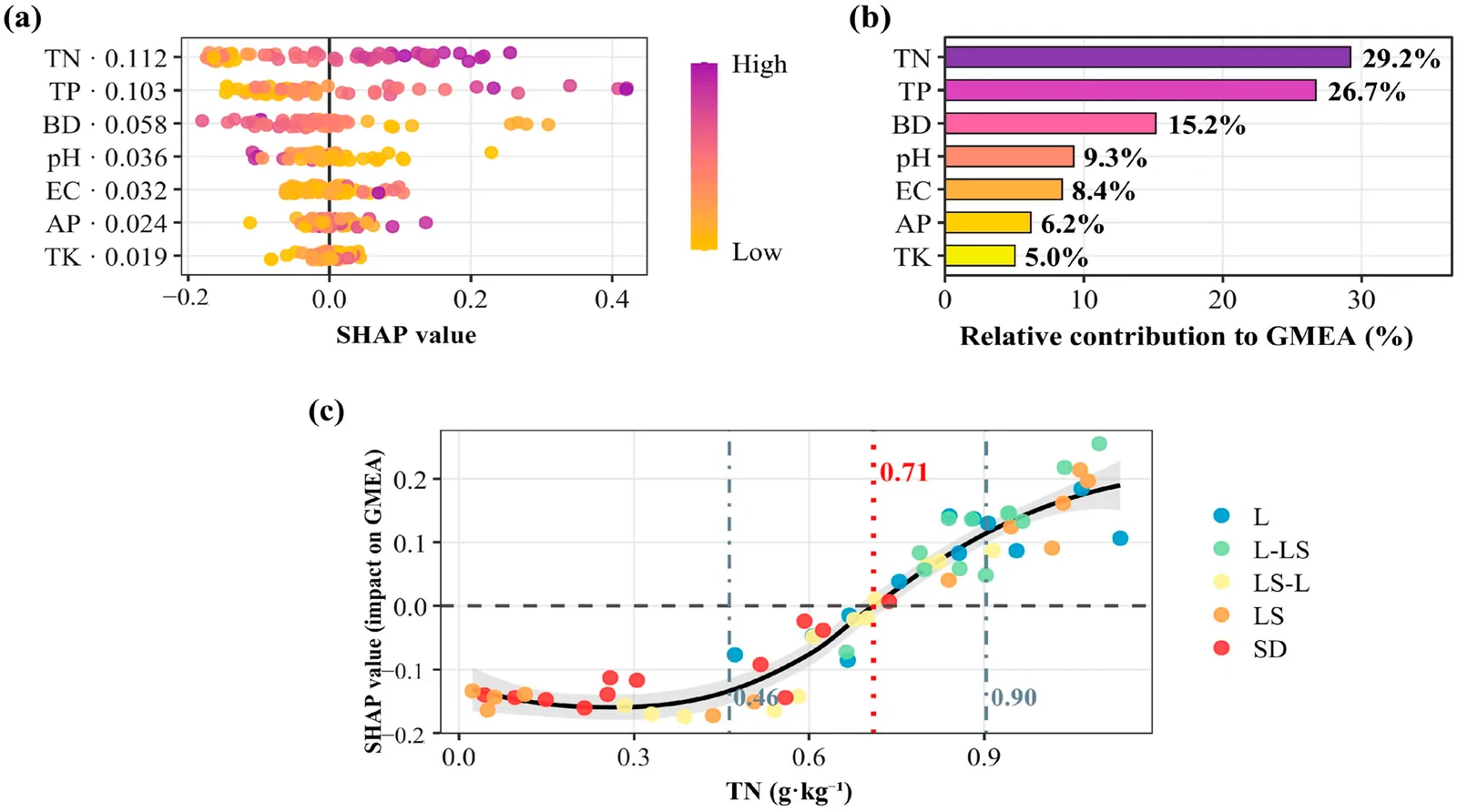
XGBoost-SHAP interpretation of geometric mean enzyme activity (GMEA). (**a**) SHAP summary plot; (**b**) relative predictive importance based on normalized mean absolute SHAP values; and (**c**) SHAP dependence plot for TN with a LOESS fit and 95% confidence interval. SHAP values indicate positive or negative contributions to predicted GMEA relative to the model baseline. The red line marks the zero-crossing transition point, and blue-gray lines indicate the 25th and 75th percentiles of TN.

**Figure A1:**
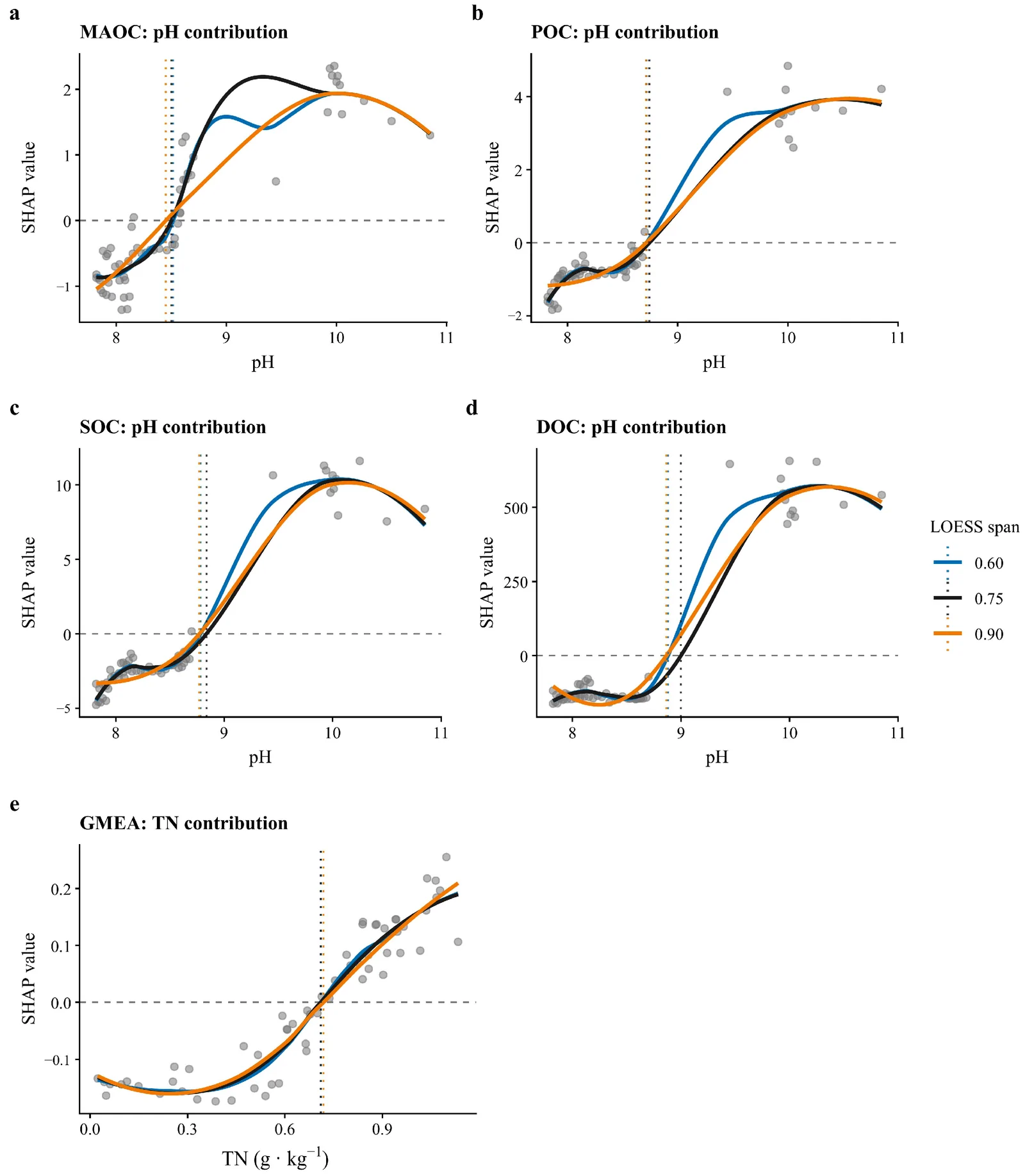
Sensitivity of SHAP response transition points to LOESS span. Curves represent LOESS fits with spans of 0.60, 0.75, and 0.90; dotted vertical lines mark the corresponding zero crossings. Points are sample-level SHAP values from the fixed XGBoost models. (**a**) MAOC; (**b**) POC; (**c**) SOC; (**d**) DOC; (**e**) GMEA.

In the GMEA model, TN and TP contributed 29.2% and 26.7%, respectively, together accounting for 55.9% of relative predictive importance (Figure 7b). The fitted TN contribution to GMEA changed from negative to positive at approximately 0.71 g·kg^−1^ (Figure 7c). By comparison, GMEA contributed only 4.2–8.0% in the four carbon-fraction models. Predictor rankings therefore differed between the carbon fractions and GMEA. Across LOESS spans of 0.60, 0.75, and 0.90, the estimated pH transition points ranged from 8.45 to 8.52 for MAOC and from 8.71 to 8.74 for POC. The corresponding ranges were 8.77–8.84 for SOC and 8.86–9.00 for DOC. The estimated TN transition point for GMEA ranged from 0.710 to 0.719 g·kg^−1^. The response order of MAOC, POC, SOC, and DOC remained unchanged across the tested spans, although the DOC transition point showed the greatest sensitivity to the LOESS span (Figure A1).

## 4. Discussion

### 4.1. Carbon-Enzyme Association Patterns and Decoupling Across Salt-Affected Soil Types

Soil carbon transformation depends on organic substrates, nutrient availability, and extracellular enzyme activity [28,29]. The strength and direction of associations between carbon fractions and extracellular enzymes differ among the five salt-affected soil types (Figure 4).

In L soil, DOC remains positively correlated with URE, BG, and CAT after BH correction (r = 0.84 to 0.97, BH-adjusted *p* < 0.01). AP and TP are positively associated with DOC and selected enzyme activities, whereas pH and TK show mainly negative associations. L-LS soil has a broader pattern of positive internal correlations. All carbon fractions are positively correlated with one another, and several carbon-enzyme correlations involving BG, SUC, and URE remain significant. Similar covariation between carbon substrates and enzyme activities has been reported in alpine grasslands on the Qinghai-Tibet Plateau [30,31]. Substrate and nutrient availability can support microbial substrate use and extracellular enzyme production [29,31]. The correlations involving AP and TP in L soil, together with the broad positive carbon-enzyme pattern in L-LS soil, are consistent with relatively synchronous variation in carbon pools and enzyme activities.

Association directions become more variable in LS and LS-L soils. In LS soil, DOC is negatively correlated with URE and SUC, whereas MAOC is positively correlated with SUC. Soluble and mineral-associated carbon therefore show different relationships with hydrolytic enzyme activity. LS-L soil retains the largest number of significant correlations between physicochemical properties and carbon or enzyme variables, but these correlations include both positive and negative directions. Salt composition, pH, and nutrient conditions can alter substrate availability and microbial metabolism in salt-affected soils [11,13,28]. The mixed correlations in LS and LS-L soils are therefore consistent with different responses of carbon fractions and enzyme functions to multiple environmental constraints.

**Figure A2:**
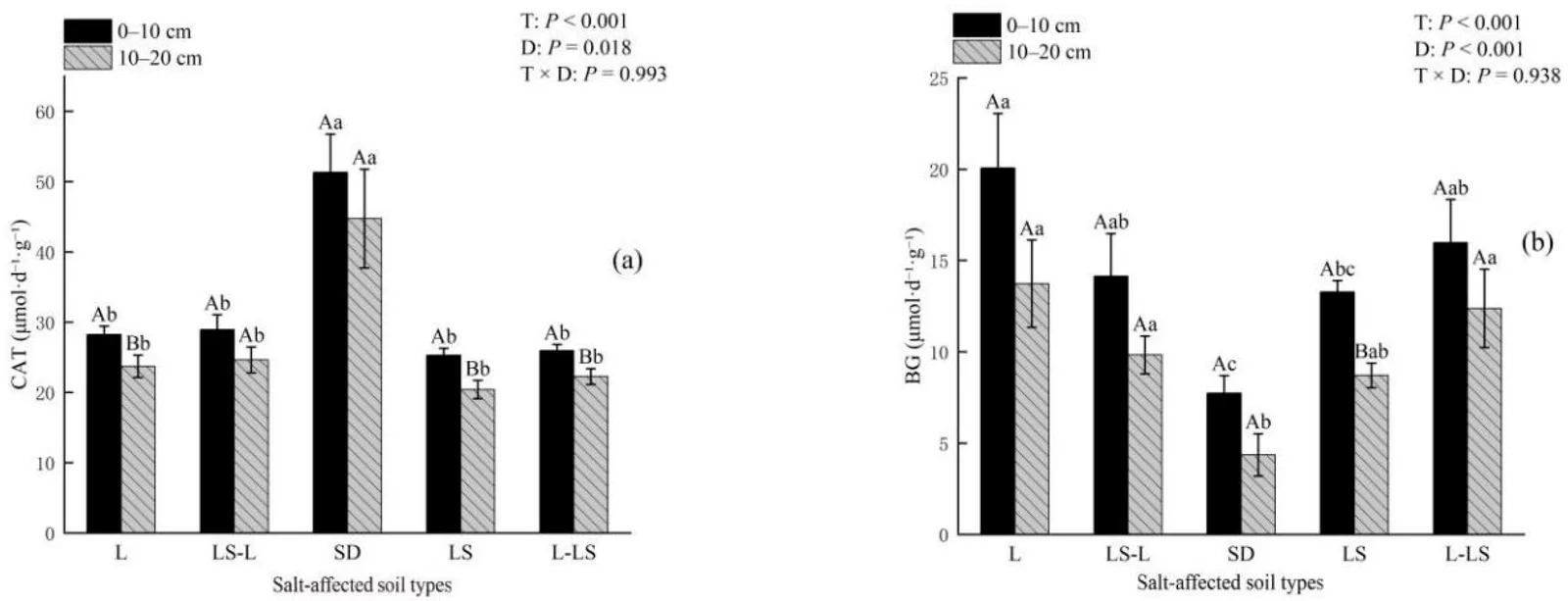

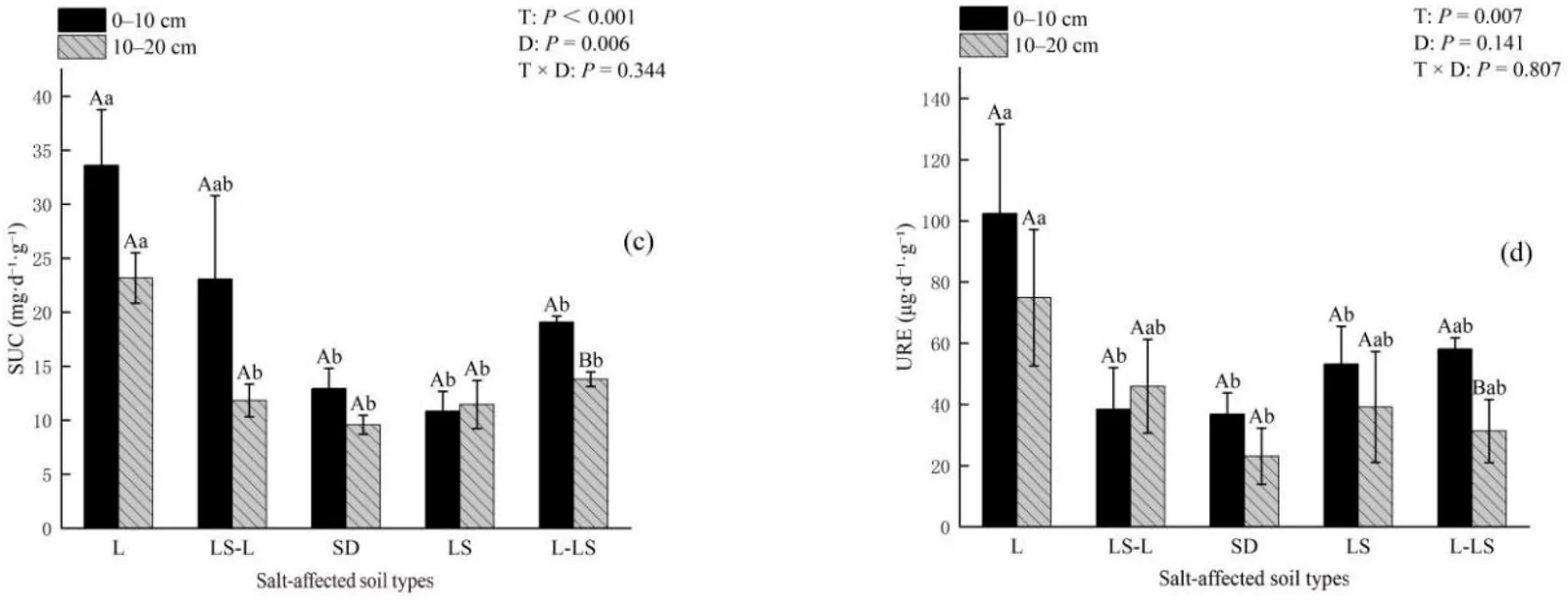
Extracellular enzyme activities across salt-affected soil types and soil depths. (**a**) Catalase activity (CAT); (**b**) β-glucosidase activity (BG); (**c**) sucrase activity (SUC); and (**d**) urease activity (URE). Bars represent the mean ± standard error. Different lowercase letters indicate significant differences among soil types within the same soil depth, whereas different uppercase letters indicate significant differences between soil depths within the same soil type (*p* < 0.05). T, salt-affected soil type; D, soil depth; T × D, interaction between soil type and soil depth.

SD soil contains the highest concentrations of all measured carbon fractions, but no significant carbon-enzyme correlation remains after BH correction. SOC and DOC remain positively correlated with each other, while carbon fractions are not significantly correlated with CAT, BG, SUC, or URE. CAT activity is relatively high in SD soil, whereas BG, SUC, and URE activities are comparatively low (Figure A2). This combination indicates reduced covariation between carbon pool size and hydrolytic enzyme activity. Soda soils commonly have high pH and substantial carbonate accumulation, which can restrict microbial substrate utilization and inhibit enzymes involved in carbon and nitrogen cycling [32,33]. The contrasting responses of CAT and the hydrolytic enzymes are consistent with their different functional sensitivities to alkaline conditions. Lower hydrolytic enzyme activities reduce the potential for enzymatic substrate decomposition and may contribute to the retention of incompletely processed carbon [20,28].

The five soil types therefore show distinct carbon-enzyme association structures. L soil has DOC-centered relationships, L-LS soil has broad positive correlations, LS and LS-L soils show directional divergence, and significant carbon-enzyme correlations are absent in SD soil after BH correction. In this study, carbon-enzyme decoupling is used operationally to describe reduced synchrony, divergence in correlation direction, or loss of significant associations between carbon fractions and enzyme activities. Salt-affected soil type is thus associated with both carbon pool composition and the organization of carbon-enzyme relationships. Because carbon mineralization and microbial resource allocation were not measured directly, the process linking lower hydrolytic enzyme activity to carbon retention in SD soil requires further experimental evaluation.

### 4.2. Nonlinear Physicochemical Responses Associated with Carbon-Enzyme Decoupling and Carbon Pool Restructuring

The XGBoost-SHAP analysis identifies different leading predictors for the carbon fractions and geometric mean enzyme activity (GMEA). pH has the highest relative predictive importance for SOC, DOC, POC, and MAOC, whereas TN and TP rank highest in the GMEA model. These contrasting predictor profiles are consistent with the asynchronous variation in carbon-pool composition and enzyme activity described in Section 4.1. Although all pairwise Spearman correlations among predictors are below 0.70, this does not eliminate covariance among pH, EC, and nutrient conditions along the soil-type gradient. SHAP importance therefore represents the contribution of pH conditional on the other predictors and should not be interpreted as an isolated causal effect. In this dataset, pH may partly represent the broader alkaline environment associated with differences among soil types.

As pH increases, the SHAP values for MAOC, POC, SOC, and DOC cross zero at approximately 8.50, 8.74, 8.84, and 9.00, respectively. These zero crossings define response transition points at which the direction of the fitted pH contribution changes. The contribution becomes positive first for MAOC and last for DOC, indicating fraction-specific response ranges along the alkalinity gradient. This sequence may reflect differences in mineral protection and microbial accessibility. Previous studies show that increasing pH can weaken the association of some organic matter with soil minerals [34,35], whereas carbonate accumulation may further alter mineral retention [36,37]. These changes may affect the formation and persistence of MAOC. Osmotic and ionic stress can also restrict microbial metabolism and carbon utilization, allowing released or incompletely utilized organic carbon to remain in soil [13,20,38,39]. Differences in mineral protection and microbial accessibility therefore provide a plausible explanation for the staggered responses of the carbon fractions.

GMEA shows a different relationship with environmental conditions and is more closely associated with nutrient status. The SHAP value for TN crosses zero at approximately 0.71 g·kg^−1^, with a predominantly negative fitted contribution below this value and a positive contribution above it. Below 0.46 g·kg^−1^, the negative contribution changes little further within the observed TN range. This plateau applies to the fitted SHAP contribution rather than directly to measured GMEA and indicates little additional modeled change in the TN contribution at the lowest concentrations. Together with the high predictive importance of TN and TP, this pattern is consistent with nutrient constraints on enzyme activity. By comparison, pH is more closely associated with carbon fraction distribution. High pH may alter mineral protection, whereas nutrient limitation may restrict microbial enzyme production. Microbial carbon-use efficiency describes the allocation of assimilated carbon between growth and respiration and is closely associated with SOC storage at the global scale [40]. Although carbon-use efficiency was not measured, the association between GMEA and TN provides an indirect link between nutrient status and microbial carbon processing. The different responses of carbon fractions and GMEA are therefore consistent with carbon-enzyme decoupling.

The PCoA results show partial overlap among the L, L-LS, LS-L, and LS samples, whereas the SD samples are clearly separated in the ordination space (Figure 3a). This separation coincides with the high pH and low TN of SD soil and is consistent with the nonlinear patterns identified by SHAP. Ecological processes may respond nonlinearly when environmental stress approaches or exceeds the tolerance range of biological communities [3,41]. Global soil analyses also identify context-dependent microbial thresholds associated with pH, nitrogen, and temperature [15]. The distinct position of SD soil is therefore consistent with altered carbon fraction distribution under concurrent alkaline and nutrient constraints.

The relative distributions of the measured carbon fractions further support this interpretation (Figure 3b). MAOC is the dominant measured carbon fraction in L, L-LS, and LS-L soils. Its relative contribution decreases in LS soil as that of POC increases. Within the measured carbon-fraction pool, SD soil has the lowest MAOC proportion and the highest POC and DOC proportions. Although SD soil contains the highest absolute MAOC concentration, MAOC makes the smallest relative contribution to the summed measured fractions because POC and DOC contribute larger shares. Absolute concentration and relative contribution therefore describe different aspects of carbon-pool structure. This distinction reconciles the positive fitted pH contribution to absolute MAOC with its low relative contribution in SD soil. Weaker mineral retention at high pH [34,35,36,37] and reduced microbial utilization under low TN [7,42] may favor the relative retention of POC and DOC.

The contrasting predictor sets and response transition points associate carbon fraction distribution mainly with pH and GMEA mainly with nutrient status, particularly TN. This separation is consistent with the distinct carbon-pool composition of SD soil, where no carbon-enzyme correlations remain significant after Benjamini-Hochberg correction. The reported transition points are point estimates derived from LOESS fits to 60 samples. Their 95% confidence bands describe uncertainty in the fitted curves but do not provide confidence intervals for the crossing locations. The pH values of 8.50 to 9.00 and the TN value of 0.71 g·kg^−1^ should therefore be interpreted as approximate regional transition points within the sampled environmental range. They provide quantitative reference points for the Qiangtang Plateau but are not universal ecological or management thresholds. Their applicability to other alpine soils requires evaluation using independent samples and controlled experiments.

### 4.3. Ecological Implications of Carbon-Enzyme Decoupling and Risks for Carbon Sink Assessment

Soil carbon stocks do not necessarily indicate long-term carbon pool stability [7]. Under conditions characterized by carbon-enzyme decoupling, limited microbial utilization may allow organic carbon to remain in relatively labile fractions such as POC and DOC. Compared with MAOC, these fractions generally turn over faster and are more sensitive to changes in temperature, moisture, and microbial activity [42,43]. High SOC concentrations in salt-affected soils may therefore reflect either stable carbon storage or temporary retention of labile carbon under restricted decomposition. These two pathways have different implications for long-term carbon stability.

Studies in Tibetan alpine ecosystems show that warming and altered precipitation affect soil water availability and microbial community composition [44,45]. Whole-soil warming can also increase soil carbon emissions [46]. If changing temperature or moisture conditions relieve constraints on microbial activity, retained POC and DOC may re-enter decomposition and increase the risk of short-term CO_2_ release. Greater precipitation may also enhance DOC transport and redistribute carbon within surface soils [43]. The persistence of labile carbon under saline and alkaline stress thus depends on subsequent changes in temperature, moisture, microbial activity, and mineral protection. Under continued warming and changing precipitation regimes, salt-affected soils with high proportions of labile carbon may be more susceptible to carbon loss [42,46].

**Figure 8:**
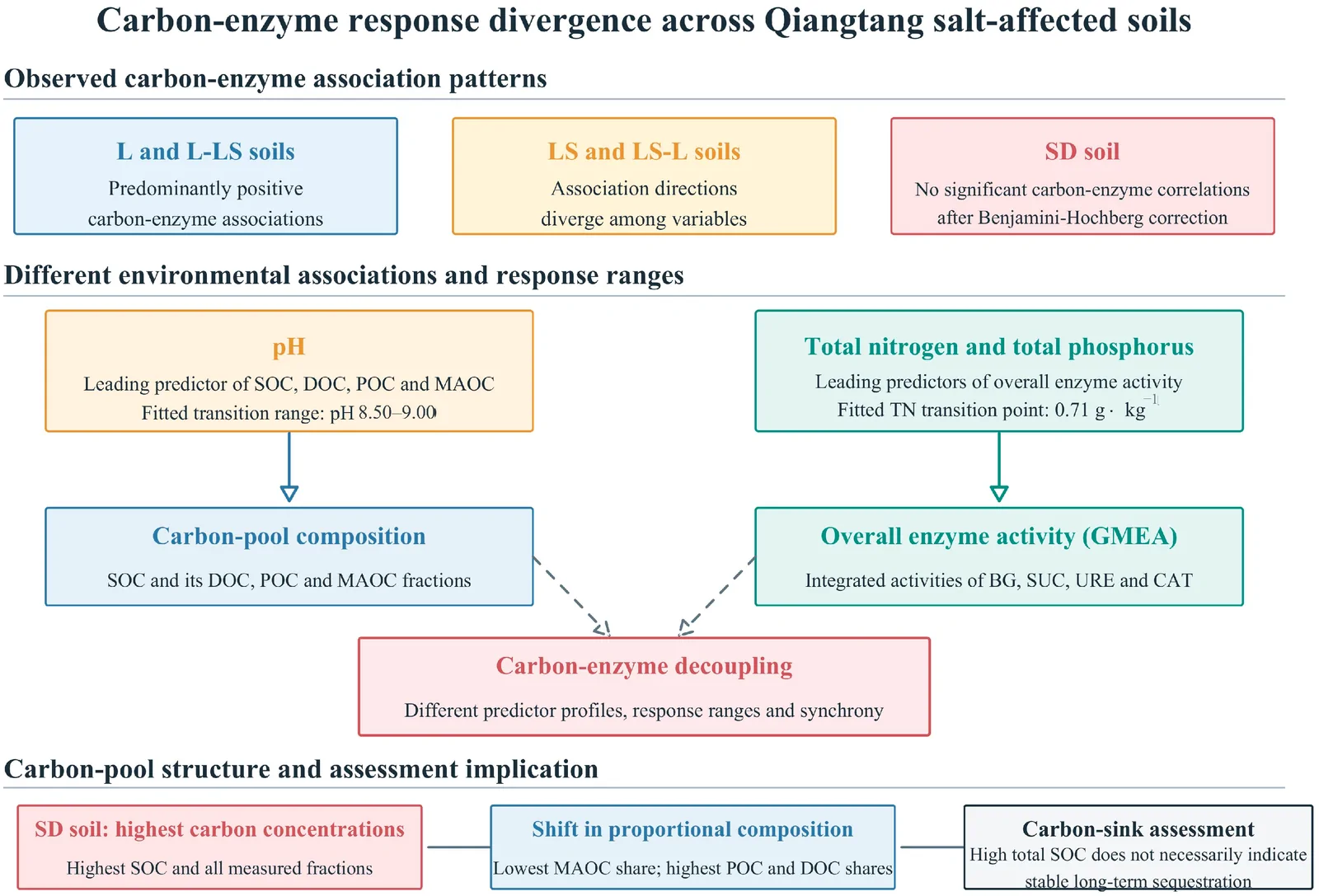
Conceptual summary of carbon–enzyme decoupling in salt-affected soils of the Qiangtang Plateau. Carbon–enzyme associations are predominantly positive in L and L–LS soils, diverge in LS and LS–L soils, and are not significant in SD soil after Benjamini–Hochberg correction. Carbon fractions are associated mainly with pH, whereas GMEA is associated mainly with TN and TP. SD soil combines high carbon concentrations with a low MAOC proportion and high POC and DOC proportions. Arrows indicate predictive or interpretive relationships, not demonstrated causality.

Carbon sink assessments for alpine salt-affected soils need to distinguish current carbon stocks from the capacity for sustained net carbon sequestration. Reliance on total SOC alone may lead to carbon retained under restricted decomposition being interpreted as stable carbon sequestration. A more informative assessment would combine the relative proportions of DOC, POC, and MAOC with extracellular enzyme activities and carbon-enzyme associations. Measurements of pH, nutrient conditions, and CO_2_ fluxes would provide additional information on environmental constraints and carbon exchange. This approach would help identify soils with high carbon stocks but large labile fractions and relatively low long-term stability, thereby improving estimates of carbon sink capacity and carbon loss risk. The relationships among soil type, carbon-pool composition, enzyme activity, and their main environmental predictors are summarized conceptually in Figure 8.

This study is based on a single spatial survey comprising two sites per soil type and three quadrats nested within each site. Although this design captures within-site heterogeneity, site-level replication is limited, and the analyses do not explicitly model clustering among observations from the same site. The statistical and ecological interpretations therefore describe patterns within the sampled Qiangtang sites rather than region-wide estimates. Future studies should increase independent site replication and combine hierarchical statistical models and site-blocked model validation with long-term field monitoring and controlled manipulations of temperature, moisture, salinity, pH, and nutrient supply. Concurrent measurements of carbon fractions, extracellular enzyme activities, microbial communities, and CO_2_ fluxes would clarify how retained labile carbon responds to changes in climate and soil water-salt conditions and improve assessments of long-term carbon pool stability in alpine salt-affected soils.

## 5. Conclusions

Carbon–enzyme association patterns vary among the five salt-affected soil types. In L soil, these relationships center on DOC, which is positively correlated with URE, BG, and CAT. L–LS soil exhibits a broader range of positive correlations, whereas associations in LS and LS–L soils vary in direction. After Benjamini–Hochberg adjustment, no significant carbon–enzyme correlations remain in SD soil. This absence is consistent with reduced synchrony between organic carbon fractions and extracellular enzyme activities under SD conditions. Carbon–enzyme decoupling is therefore expressed as divergence in correlation direction, reduced synchrony, or loss of significant correlations, rather than a uniform weakening of all relationships.

Carbon fractions and GMEA also differ in their leading predictors and response transition points. pH has the greatest relative predictive importance for SOC, DOC, POC, and MAOC, accounting for 36.1% to 48.7% of model importance. TN and TP rank highest in the GMEA model, with contributions of 29.2% and 26.7%, respectively. By comparison, GMEA contributes only 4.2% to 8.0% of predictive importance in the carbon-fraction models. The fitted pH contributions to the carbon fractions change from negative to positive between 8.50 and 9.00. The fitted TN contribution to GMEA changes direction at 0.71 g·kg^−1^ and varies little below 0.46 g·kg^−1^. The order of the carbon-fraction transitions and the TN transition point remains stable across the tested LOESS spans. These results indicate that carbon pool composition and enzyme activity have different environmental associations.

Soil depth significantly affects SOC, DOC, and POC but has no significant overall effect on MAOC. SD soil contains the highest concentrations of SOC and all measured carbon fractions, although its carbon pool composition differs from those of the other soil types. Within the measured carbon-fraction pool, MAOC accounts for 39.41%, whereas POC and DOC account for 54.23% and 6.36%, respectively. PCoA separates SD soil from the other soil types, and PERMANOVA confirms overall differences in carbon pool composition. Thus, the high organic carbon concentration in SD soil is accompanied by greater relative contributions of particulate and dissolved carbon and a lower contribution of mineral-associated carbon. This composition does not necessarily indicate greater long-term carbon stability. Because POC and DOC are relatively sensitive to changes in temperature, moisture, and microbial activity, their increased contributions may raise the risk of carbon loss under environmental fluctuations.

Carbon sink assessments for alpine salt-affected soils should consider carbon stocks, carbon pool composition, and carbon–enzyme association patterns together. Total SOC alone cannot distinguish mineral-protected carbon from labile carbon retained under limited decomposition and may therefore overestimate long-term carbon sink potential. The pH and TN response transition points provide empirical reference points for the sampled Qiangtang sites within the observed environmental range. These transition points are model-dependent features of the present dataset and should not be regarded as universal ecological thresholds.

## Figures and Tables

**Table 1:** Vegetation characteristics and major water-soluble anion contents of sampling sites representing different salt-affected soil types.

Salt-Affected Soil Type	Geographic Coordinates	Dominant Species	Vegetation Cover (%)	Cl^−^ (mg·g^−1^)	S0_4_^2−^ (mg·g^−1^)	C0_3_^2−^ (mg·g^−1^)	Cl^−^/S0_4_^2−^ Equivalent Ratio
L	33°13′52.60″ N 90°23′27.96″ E	*Rhodiola quadrifida*	55	0.242	0.050	0.010	6.54
	34°38′27.68″ N 88°35′59.18″ E	*Carex moorcroftii*	45	0.179	0.055	0.016	4.4
LS-L	33°11′23.52″ N 88°19′47.48″ E	*Carex moorcroftii* *Stipa purpurea*	30	0.329	0.296	0.010	1.5
	34°25′31.60″ N 88°35′29.84″ E	*Carex moorcroftii*	20	0.777	0.621	0.019	1.69
SD	32°59′52.03″ N 86°52′46.43″ E	*Stipa purpurea*	20	0.120	0.152	0.224	1.07
	32°58′27.26″ N 86°43′35.45″ E	*Carex moorcroftii* *Stipa purpurea*	25	0.122	0.146	0.231	1.13
LS	32°59′52.45″ N 86°52′48.13″ E	*Stipa purpurea*	40	0.665	12.793	0.009	0.07
	34°38′27.38″ N 88°35′59.34″ E	*Carex moorcroftii*	35	0.623	12.766	0.011	0.07
L-LS	34°27′23.71″ N 88°41′09.21″ E	*Carex moorcroftii* *Potentilla fruticosa*	35	0.060	0.108	0.015	0.75
	33°52′58.72″ N 88°21′28.97″ E	*Myricaria prostrata* *Kobresia pygmaea*	55	0.047	0.098	0.015	0.65

Note: EC was measured in a 1:5 soil-to-water extract; therefore, ECe-based salinity severity classes were not assigned.

## Data Availability

The data presented in this study are available on request from the Corresponding author due to privacy or ethical restrictions.

## Appendix A

**Table A1:** Cross-validated predictive performance of the XGBoost regression models.

Response Variable	CV-R^2^	CV-RMSE
GMEA	0.4094	0.2835
SOC	0.7956	3.4805
DOC	0.8815	155.6734
POC	0.6571	1.8288
MAOC	0.5340	1.3958

CV-R^2^, cross-validated coefficient of determination; CV-RMSE, cross-validated root mean square error. Values were obtained using fivefold cross-validation with a random seed of 42.

**Table A2:** Physicochemical properties of different salt-affected soil types at two soil depths.

Salt-Affected Soil Type	Soil Depth (cm)	pH	BD (g·cm^−3^)	AP (mg·kg^−1^)	TP (g·kg^−1^)	TK (g·kg^−1^)	TN (g·kg^−1^)
L	0–10	8.12 ± 0.12b	1.56 ± 0.11a	8.08 ± 1.48a	0.53 ± 0.05a	3.86 ± 0.69a	0.92 ± 0.03ab
	10–20	8.27 ± 0.11b	1.48 ± 0.07b	7.59 ± 1.18a	0.51 ± 0.03a	8.10 ± 2.50ab	0.72 ± 0.09a
LS-L	0–10	8.19 ± 0.16b	1.56 ± 0.03a	8.21 ± 1.71a	0.44 ± 0.08a	9.20 ± 3.13a	0.69 ± 0.08abc
	10–20	8.26 ± 0.16b	1.52 ± 0.05ab	6.25 ± 1.05ab	0.37 ± 0.04a	11.36 ± 4.15a	0.54 ± 0.08a
SD	0–10	9.92 ± 0.11a	1.57 ± 0.03a	8.99 ± 0.67a	0.26 ± 0.03b	3.48 ± 0.67a	0.47 ± 0.10c
	10–20	10.24 ± 0.15a	1.56 ± 0.04ab	7.06 ± 0.62ab	0.23 ± 0.01b	2.88 ± 0.59b	0.25 ± 0.07b
LS	0–10	8.34 ± 0.12b	1.56 ± 0.06a	7.96 ± 0.21a	0.29 ± 0.04b	6.30 ± 2.89a	0.56 ± 0.22bc
	10–20	8.41 ± 0.12b	1.62 ± 0.03ab	6.76 ± 0.61ab	0.38 ± 0.09a	4.56 ± 1.56ab	0.63 ± 0.15a
L-LS	0–10	8.03 ± 0.05b	1.65 ± 0.03a	5.74 ± 0.34a	0.39 ± 0.02ab	6.42 ± 1.68a	0.96 ± 0.04a
	10–20	8.15 ± 0.06b	1.69 ± 0.02a	4.59 ± 0.70b	0.41 ± 0.02a	8.23 ± 1.44ab	0.83 ± 0.04a

Values are means ± standard errors. Different lowercase superscript letters within the same column and soil depth indicate significant differences among salt-affected soils (*p* < 0.05). L, chloride type; LS-L, sulfate-chloride type; SD, soda type; LS, sulfate type; L-LS, chloride-sulfate type. BD, soil bulk density; AP, available phosphorus; TP, total phosphorus; TK, total potassium; TN, total nitrogen.
